# Aneurysmatic subarachnoid haemorrhage

**DOI:** 10.1186/s42466-019-0015-3

**Published:** 2019-04-29

**Authors:** Oezguer A. Onur, Gereon R. Fink, Joji B. Kuramatsu, Stefan Schwab

**Affiliations:** 1 Department of Neurology, University Hospital, University of Cologne, Kerpener Str, 62 50937 Köln, Germany; 2 Department of Neurology, University Hospital, University of Erlangen, Erlangen, Germany

**Keywords:** Standard operating procedure, Vasospasm, Clipping, Coiling, Nimodipine, Delayed cerebral ischaemia

## Abstract

**Introduction:**

Aneurysmatic Subarachnoid Haemorrhage (aSAH) is typically caused by extravasated blood in the subarachnoid space due to a ruptured aneurysm. aSAH is often life-threatening in the acute stage, but may also cause secondary brain damage due to delayed cerebral ischaemia (DCI) and other complications in the days and weeks after the initial bleeding. Rapid onset of a most severe headache is a typical sign of a non-traumatic aSAH besides a reduced level of consciousness and neurologic deficits.

**First steps:**

Immediate diagnostic steps in case of a suspected SAH are cerebral imaging (CCT, MRI) and lumbar puncture. If a SAH is confirmed, a digital subtraction angiography should be performed to detect an aneurysm. If an aneurysm is detected it should be occluded immediately after interdisciplinary consultation with neurosurgeons and neuroradiologists.

**Comments:**

If endovascular coiling and surgical clipping are both available and equally suitable, coiling should be preferred due to a better long-time outcome. Often the age of the patient, the location of the aneurysm, and the configuration of the aneurysm result in favouring one or the other technique. Special care aims at avoiding stress, increased intracranial pressure, pain, fever, emesis, and at keeping glucose levels and electrolytes in the normal range. As nimodipine is associated with a better outcome, it should be administered from the beginning. To detect vasospasm, serial transcranial doppler should be performed at least once a day for at least 14 days. If vasospasms are detected, this procedure needs to be continued until flow velocity returns to the normal range. To detect an increased intracranial pressure, external ventricular drainage or intraparenchymal probes are recommended. Regarding haemodynamics, euvolaemia and normotension should be achieved. If vasospasms and/or an increased intracranial pressure occur, mean arterial pressure needs to be adjusted to ensure an adequate cerebral perfusion pressure.

**Conclusions:**

If immediate actions are taken to treat the aneurysm and complications in the following weeks are handled with care, a favourable outcome is possible for this otherwise often devastating disease.

## Introduction

**Fig. 1:**
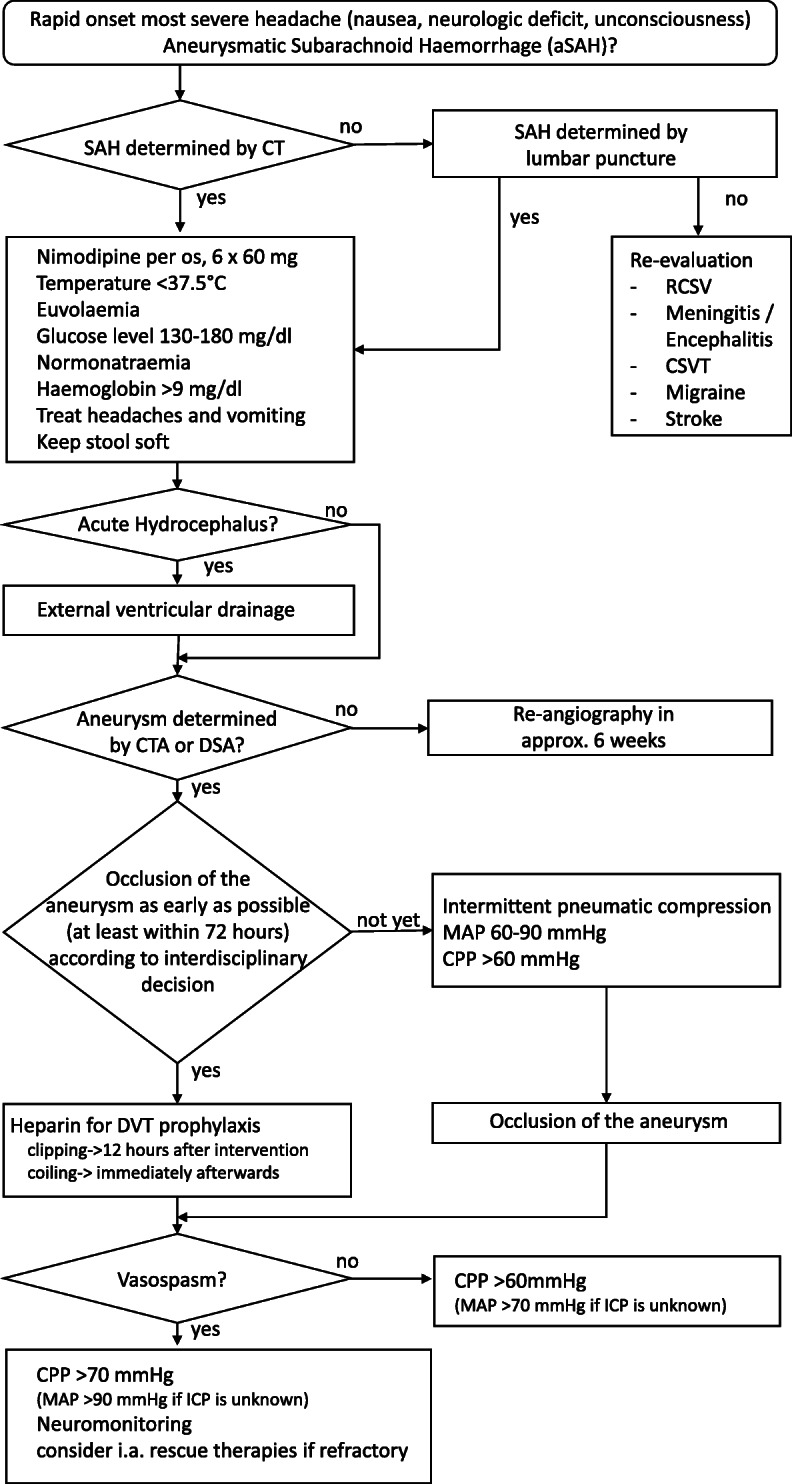
Flow chart for the treatment of aneurysmatic subarachnoid haemorrhage

An aneurysmatic Subarachnoid Haemorrhage (aSAH) is a life-threatening disease with a mortality rate of 25–50% [4] in which blood extravasates from a ruptured aneurysm into the subarachnoid space and basal cisterns but may also penetrate cerebral tissue (Fig. 1). Besides of an aneurysm, other causes for SAH are venous bleeding (perimesencephalic SAH), trauma, and SAH following primary intracerebral bleeding, venous sinus thrombosis, coagulopathy, dissection of intracranial vessels, cerebral amyloid angiopathy, cerebral vasoconstriction, cocaine.

Typically, patients report a most severe headache (“worst ever”, “thunderclap headache”) with a sudden onset. This is often accompanied by neck stiffness, nausea, vomiting, decreased level of consciousness, vegetative disturbances (e.g., sweating, cardiac arrhythmias, electrocardiographic changes), neurologic deficits (oculomotor palsy, hemiparesis, papilledema), and epileptic seizures [1]. An aSAH often occurs after physical exercise [1].

In 10 to 43% of the patients, a warning leak (“sentinel headache”, i.e. minor haemorrhage) can occur up to eight weeks before the aSAH [1].

For grading, various scores are used (see Tables 1 and 2). A recent score to prognosticate cognition and quality of life is the Functional Recovery Expected after Subarachnoid Haemorrhage (FRESH). This score encompasses in a multidimensional manner the Hunt & Hess grading, APACHE II (“Acute Physiology And Chronic Health Evaluation II”) physiologic scores on admission, age, and an aneurysmal rebleed within 48 h [13].

**Table 1:** Clinical Grading as defined by the World Federation of Neurological Surgeons (WFNS), Hunt & Hess and in relation to the Glasgow Coma Scale (GCS)

Grades WFNS/Hunt & Hess	Symptoms described by Hunt & Hess	WFNS	
		GCS	hemiparesis/aphasia
I	asymptomatic, or minimal headache and neck stiffness	15	absent
II	severe headache, neck stiffness, cranial nerve palsy (optional)	13–14	absent
III	drowsiness, minimal neurologic deficit	13–14	absent
IV	stupor, severe neurologic deficit (hemiparesis), vegetative disturbances	7–12	present or absent
V	coma, signs of decerebration	3–6	present or absent

**Table 2:** Grading based on CT-scan as defined by the (modified) Fisher scale and associated risk for delayed cerebral ischaemia (DCI)

Grade	modified Fisher scale	DCI risk
0	absent SAH or intraventricular haemorrhage	minimal (0%)
1	focal or diffuse, thin (< 1 mm) SAH, no intraventricular haemorrhage	low (24%)
2	focal or diffuse, thin (< 1 mm) SAH, present intraventricular haemorrhage	moderate (33%)
3	focal or diffuse, thick (> 1 mm) SAH, no intraventricular haemorrhage	moderate (33%)
4	focal or diffuse, thick (> 1 mm) SAH, present intraventricular haemorrhage	high (40%)

### First steps

Multicenter randomized controlled trials assessing the different aspects of the management of aSAH are sparse (or even lacking). Accordingly, evidence-based recommendations are often difficult to make. However, decisions need to be taken in handling patients with aSAH, therefore this SOP is based on guidelines and, where available, on clinical trials.

If clinical signs and symptoms are suggestive for an aSAH immediate cerebral imaging is essential, preferably CT (sensitivity 95%) including CT angiography. If cerebral imaging shows no signs of bleeding, lumbar puncture must be performed [1, 10] to check the following parameters:

- visual inspection
  - bloody cerebrospinal fluid (consider artificial blood due to traumatic tap)
  - xanthochromia
- laboratory work-up
  - erythrocytes in the cerebrospinal fluid
  - xanthochromia
  - ferritine level
  - siderophages

Sensitivity is higher if the lumbar puncture is performed 8–12 h after the first symptoms [10, 11].

If the patient’s condition is unstable concerning the cardiopulmonary system, sedation and intubation have to be initiated first (preferably with Midazolam/Ketamine). Patients should be treated on neurological or neurosurgical intensive care units [2]. Mild cases (H + H/WFNS Grad 1, Table 1) may be admitted to stroke units [11].

Digital subtraction angiography (DSA) should be performed within 24 h after the initial bleeding (gold standard). If logistically possible and suitable, aneurysm occlusion by coiling can be performed in the same session. If no aneurysm is detected, a re-angiography after the period of (possible) vasospasm is recommended [10, 11].

The highest incidence of re-bleeding is during the first 72 h (4–14% on day 1, the following days approx. 1–2% per day for one month). Accordingly, an immediate interdisciplinary diagnostic work-up and planning of interventional procedures together with neuroradiologists and neurosurgeons is recommended [1, 10, 11]. If both procedures (coiling and clipping) are equally available and suitable, coiling is preferable due to a better long-time outcome [1, 6–8, 10–12] and better cognitive outcome [9].

Findings and features favouring clipping [1, 10]:

- Location: middle cerebral and pericallosal artery
- Configuration of the aneurysm: wide neck of the aneurysm, arterial branches stemming from the aneurysm
- Relevant intracranial haemorrhage

Findings and features favouring coiling [1, 10]:

- Location: posterior circulation
- Configuration of aneurysm: small neck of aneurysm
- Patient age above 70 years
- poor-grade aSAH based on WFNS-scoring

Occlusion of the aneurysm via endovascular coiling or surgical clipping [12] should be performed as early as possible but no later than 72 h after aSAH [10].

If a hydrocephalus is present, external ventricular drainage should be applied prior to aneurysm occlusion (avoid aggressive drainage) [11].

### General management in the emergency department, stroke unit, and ICU

Patients should rest in bed [11] in sitting-up head positioning (≥30°). Central venous and arterial line should be placed, whenever needed (if a femoral placement is considered, the right femoral artery should be reserved for angiography; CAVE: arterial line transducer should be placed at the level of the foramen of Monroi).

Serial neurological assessment and neurologic monitoring should be performed regularly. Headaches should be treated (aim at a VAS or Behavioural Pain Scale ≤4) using analgesics [10], e.g., Novalminsulfon and Piritramid (avoid Tramadol because of nausea, a decrease of the seizure threshold, and drowsiness). If antiemetic drugs are needed, Setrons should be preferred and Dimenhydrinat avoided due to a decreased level of consciousness. Stool should be kept soft using laxatives [10, 11], preferably with Lactulose.

Fever (temperature above 37.4 °C) should be treated medically and physically [1, 2, 10, 11]; surface cooling or intravascular devices (Thermogard) can be used [1, 2]. If tolerable, forced normothermia (36.5 °C) should be considered. During the period of severe vasospasm mild hypothermia should be considered (35°-36 °C).

Regarding blood glucose, volume, and electrolyte management, euvolaemia [1, 11] and glucose levels of 130–180 mg/dl should be achieved ([1, 10]; [2]). Isotonic crystalloid should be preferred if volume replacement is needed ([2]). Hypo- or hypernatraemia should be avoided [11]. In the case of hyponatraemia, fluid restriction is unfavourable since fluid restriction increases the risk of DCI [11]. Anaemia should be avoided [1], haemoglobin levels should be kept above 9 g/l [2].

For deep venous thrombosis prophylaxis, sequential compression stockings should be used before occlusion of the aneurysm [2, 10]. Immediately after the occlusion of the aneurysm by coiling, thrombosis prophylaxis should be performed by low molecular weight heparin or unfractionated heparin [2, 11]. After occlusion of the aneurysm by clipping, thrombosis prophylaxis via low molecular weight heparin should be started 12 h after the surgical procedure [10].

### Haemodynamic monitoring and blood pressure management

The treatment depends on the intracranial pressure (ICP) and the presence of vasospasm. The recently published HIMALAIA-trial showed no favorable outcome for induced hypertonia up to 130 mmHg mean arterial pressure [3]. However, in this study the mean arterial pressure in the no intervention group was at least 80 mmHg, and above 100 mmHg for approximately half of the period of the investigation. Further, a relevant proportion of the patients in the control group received vasopressors.

The following recommendations can be made:

Untreated aneurysm, normal ICP:

- systolic blood pressure < 140 mmHg
- mean arterial pressure 60–90 mmHg
- cerebral perfusion pressure > 60 mmHg [11]

Treated aneurysm, normal ICP, no vasospasm:

- normotension, CAVE: systolic blood pressure should always be above 120 mmHg, light hypertension is tolerable.

Treated aneurysm, increased ICP, no vasospasm:

- A cerebral perfusion pressure above 60 mmHg [11] and strict normocarbia (PaCO2 < 40 mmHg) are recommended.

Treated aneurysm, normal ICP, vasospasm:

- Mean arterial pressure should be increased up to > 90 mmHg stepwise (CPP > 70 mmHg), in cases of severe vasospasm even higher. Light hypercarbia should be considered to make use of vasodilatation effects, acidosis (pH < 7.2) should be avoided.

Treated aneurysm, increased ICP, vasospasm:

- Cerebral perfusion pressure should be above 70 mmHg. If cerebral blood flow is diminished, an even higher cerebral perfusion pressure should be considered. Strict normocarbia (PaCO2 35 mmHg) is recommended. Invasive neuromonitoring should be considered.

Catecholamines should be used to increase blood pressure and cerebral perfusion pressure. Severe hypervolaemia and haemodilution should be avoided, light hypervolaemia is tolerable. Under unstable conditions and the urge for increasing the catecholamines, monitoring should be extended [1], e.g., apply PiCCO.

As myocardial injury may occur in the course of an aSAH, cardiac assessment by electrocardiography, serial enzymes, and echocardiography is recommended. Cardiac output should be monitored if signs of dysfunction are detected [2, 11].

### Neurological monitoring and management of vasospasm and delayed cerebral ischaemia

A narrowing of cerebral arteries (i.e., a vasospasm) is a common complication after aSAH. Vasospasms occur most frequently seven to ten days after the haemorrhage and can last up to 21 days. Vasospasm can result in delayed cerebral ischaemia (DCI), however, not all cases of vasospasm lead to DCI, and DCI can occur without the presence of vasospasm [1]. The pathophysiology of vasospasm is not fully understood, therefore, preventive or therapeutic options remain sparse.

To detect vasospasm, serial transcranial doppler / transcranial color-coded duplex ultrasonography (TCD) at least once a day is recommended [1]. If no vasospasm occurs, daily TCD should be continued for at least 14 days. If a vasospasm is detected, TCD needs to be continued until blood flow velocity returns to the normal range. A mean blood flow velocity > 200 cm/s [2], an increase of the mean blood flow velocity more than 50 cm/s within 24 h, or an “hemispheric index” > 3 (ratio of intra- and extracranial mean blood velocity, MCA/ICA) are suggestive of a vasospasm [11]. Besides, CT/MRI perfusion and angiography may be useful for confirmation [1, 11]. However, the gold standard for detecting an arterial narrowing is DSA [2].

Although its mechanism of action remains debated, nimodipine has been shown to improve neurological outcome after an aSAH [5]. Therefore, all patients should be administered nimodipine orally 6 × 60 mg/day (every 4 h, also during the night) from the beginning. Administration via nasogastric tube is possible, albeit with decreased efficacy [11]. If enteral application is impossible [10, 11], nimodipine can be applied intravenously (continuously, 0.2 mg/ml, start with 5 ml/h; if blood pressure does not drop significantly, increase to 10 ml/h after one hour). If hypotension occurs and remains although catecholamines are applied, nimodipine dosage should be decreased or paused. Nimodipine should be applied for 21 days [2] and then reduced stepwise.

In case of a symptomatic vasospasm and relevant hypoperfusion shown by CT perfusion, consider administration of nimodipine intraarterially or angioplasty [1, 11]. Further, hypertonia is mandatory administering norepinephrine or dobutamine. Blood pressure should be increased stepwise (aim MAP > 90 mmHg) and needs to be monitored.

In case of increased intracranial pressure, hypertonic saline continuously (145–150 mmol/L) or mannitol (10%) should be considered. If further action is needed, consider thiopental (single use or continuously). Hyperventilation is only recommended for a short period of time (PaCO_2_ not less than 30 mmHg).

Extended neuromonitoring is recommended in poor-grade SAH with increased intracranial pressure and mechanical ventilation [11]:

- Serial neurological assessment, in the acute stage once per hour, check pupillary reflex.
- External ventricular drainage or intraparenchymal probes should be used to assess intracranial pressure (cerebral perfusion pressure = mean arterial pressure – intracranial pressure). An external ventricular drainage can be used to drain cerebrospinal fluid to decease intracranial pressure (CAVE: risk of rebleeding, herniation, misplacement) and to apply fibrinolytic drugs.
- In poor-grade SAH consider intensive and invasive neuromonitoring measuring brain tissue oxygen and applying cerebral microdialysis [2, 11]. Alternative: spectroscopic procedures, use indices to assess cerebrovascular autoregulation.
- Continuous EEG may help to detect epileptic seizures or DCI [2].

### Other complications

To detect a hydrocephalus malresorptivus (in 25% of the cases, acute and/or chronic stages), frequent monitoring using clinical features, ultrasound, and CT-imaging is recommended. In the case of an occlusive hydrocephalus, disturbed cerebrospinal fluid circulation and/or global cerebral edema, an external ventricular drainage should be applied immediately [1, 10, 11]. If the third and fourth ventricle is free of blood, a (overlapping) lumbar drainage can be considered [1, 10].

Approximately 10% of the patients develop epileptic seizures and/or a status epilepticus. To detect epileptic activity (mainly non-convulsive) serial EEG or continuous EEG is recommended. Prophylactic anticonvulsive treatment is not recommended [10].

Cardiopulmonary complications are common after aSAH [2]:

- ventricular and supraventricular extrasystoles or tachycardia
- atrial fibrillation
- ST segment elevation or depression
- myocardial infarction type ECG
- elevation of troponin
- neurogenic or cardiac pulmonary oedema
- cardiac failure
- cardiogenic shock

### Long-term treatment

If neurologic and/or cognitive deficits persist, rehabilitation is warranted.

For follow-up after successful occlusion of the aneurysm without complications, MR angiography is preferred rather than digital subtraction angiography [11]. Repetitive MR angiography to detect de novo aneurysms is not recommended (cumulative 5-year-probablity of 0,75%) [11].

Patients with symptomatic chronic hydrocephalus require a ventriculoperitoneal or ventriculoatrial shunt [1, 10, 11].

In case of a familial preponderance (≥2 first-degree relatives with SAH or aneurysm), screening with MR angiography can be considered. A general screening for aneurysms cannot be recommended [10, 11].

Modifiable risk factors of aneurysm rupture are smoking, high systolic blood pressure, alcohol abuse, and drugs [1, 10, 11].

## Conclusions

Immediate action concerning diagnostic and therapeutic steps are essential in the management of aSAH. After establishing the diagnosis via cerebral imaging and lumbar puncture (if imaging is inconclusive), the detected aneurysm should be occluded by surgical clipping or radiological coiling. As relevant as the acute management is the detection and treatment of complications following aSAH including vasospasm, increased intracranial pressure, disturbed cerebrospinal fluid circulation, delayed cerebral ischaemia, cardiopulmonary complications, and neurological deficits including cognitive deficits, all of which affect the clinical outcome to a great extent.

## Abbreviations

APACHE II: Acute Physiology And Chronic Health Evaluation II
aSAH: aneurysmatic Subarachnoid Haemorrhage
CPP: cerebral perfusion pressure
CT: computed tomography
DCI: delayed cerebral ischaemia
DSA: Digital subtraction angiography
DVT: deep vein thrombosis
ECG: electrocardiography
EEG: electroencephalography
FRESH: Functional Recovery Expected after Subarachnoid Haemorrhage
GCS: Glasgow Coma Scale
H + H: Hunt & Hess
HIMALAIA: Hypertension Induction in the Management of AneurysmaL subArachnoid haemorrhage with secondary IschaemiA
ICA: internal carotid artery
ICP: intracranial pressure
ICU: intensive care unit
MCA: middle cerebral artery
MR: magnet resonance
MRI: magnet resonance imaging
PaCO_2_: partial pressure of arterial CO_2_
PiCCO: Pulse Contour Cardiac Output
SAH: Subarachnoid Haemorrhage
TCD: transcranial color-coded duplex ultrasonography
VAS: visual analogue scale
WFNS: World Federation of Neurological Surgeons
